# Concerted Suppression of STAT3 and GSK3β Is Involved in Growth Inhibition of Non-Small Cell Lung Cancer by Xanthatin

**DOI:** 10.1371/journal.pone.0081945

**Published:** 2013-11-28

**Authors:** Li Tao, Fangtian Fan, Yuping Liu, Weidong Li, Lei Zhang, Junshan Ruan, Cunsi Shen, Xiaobo Sheng, Zhijie Zhu, Aiyun Wang, Wenxing Chen, Shile Huang, Yin Lu

**Affiliations:** 1 Department of Pharmacology, College of Pharmacy, Nanjing University of Chinese Medicine, Nanjing, China; 2 Engineering Center of State Ministry of Education for Standardization of Chinese Medicine Processing, Nanjing University of Chinese Medicine, Nanjing, China; 3 Jiangsu Key Laboratory for Pharmacology and Safety Evaluation of Chinese Materia Medica, Nanjing University of Chinese Medicine, Nanjing, China; 4 Department of Biochemistry and Molecular Biology, Feist-Weiller Cancer Center, Louisiana State University Health Sciences Center, Shreveport, Louisiana, United States of America; Univ of Bradford, United Kingdom

## Abstract

Xanthatin, a sesquiterpene lactone purified from Xanthium strumarium L., possesses prominent anticancer activity. We found that disruption of GSK3β activity was essential for xanthatin to exert its anticancer properties in non-small cell lung cancer (NSCLC), concurrent with preferable suppression of constitutive activation of STAT3. Interestingly, inactivation of the two signals are two mutually exclusive events in xanthatin-induced cell death. Moreover, we surprisingly found that exposure of xanthatin failed to trigger the presumable side effect of canonical Wnt/β-Catenin followed by GSK3β inactivation. We further observed that the downregulation of STAT3 was required for xanthatin to fine-tune the risk. Thus, the discovery of xanthatin, which has ability to simultaneously orchestrate two independent signaling cascades, may have important implications for screening promising drugs in cancer therapies.

## Introduction

Glycogen synthase kinase 3β (GSK3β) has emerged as one of the most attractive therapeutic targets for the treatment of neurodegenerative diseases, and GSK3β inhibitors have been successfully applied to the clinical practice for decades [1,2]. Even though it has been widely accepted that the aberrant GSK3β-mediated functions are often related to carcinogenesis, the utilization of GSK3β antagonists in cancer therapies remains enigmatic and controversial [3]. A major concern in anti-GSK3β therapy is expected to activate Wnt/β-Catenin signaling and stabilize oncogenes thus presumably lead to tumorigenesis. In cytosol, GSK3β phosphorylates β-Catenin and targets it for ubiquitination and proteasomal degradation. Therefore, inhibition of GSK3β results in β-Catenin accumulation, subsequent translocation into the nucleus and recruitment of lymphoid enhancer factor/T-cell factor (LEF/TCF) DNA-binding-mediated oncogenic proteins transcription [4].

Lung cancer is well-known for the top leading cause of mortality worldwide [5]. The current knowledge with regard to GSK3β in lung cancer progression is based on the clinical observation that phosphorylated GSK3β (Ser 9, kinase dead) might be a good prognostic marker for the epidermal growth factor receptor (EGFR) overexpressing lung carcinoma [6]. Recent evidence has shown that inhibition of GSK3β enhances the ability of the chemopreventive drug celecoxib to downregulate anti-apoptotic protein c-FLIP [7] and sensitizes tumor necrosis factor-related apoptosis-inducing ligand (TRAIL)-induced apoptosis in non-small cell lung cancer (NSCLC) [8], suggesting that disruption of GSK3β activity can serve as an optional way to block lung cancer.

The identification of new drugs from natural products has a long and successful history. In the present work, we introduce a natural sesquiterpene lactone xanthatin [9], which is isolated from *Xanthium strumarium* L. and has prominent anticancer activity, might pharmacologically interfere with GSK3β. It has been reported that the methanol extract of *Xanthium strumarium* L. that offers major xanthatin can inhibit GSK3β activity and downregulate microphthalmia-associated transcription factor (MITF)-mediated melanogenesis, while MITF is a main target of the Wnt signaling pathway [10]. These findings preliminarily suggest that there could be no causal linkage between GSK3β inhibition and Wnt activation by the plant. Moreover, If Wnt signaling activation is an inevitable outcome accompanied by GSK3β inhibition, we postulated that there could quite possibly be some preventive remedies for the risk by xanthatin. In this case, the multi-talented kinase as a therapeutic target will be realized and the utility of xanthatin will also be appreciated.

Previously, we demonstrated that xanthatin significantly induced cell cycle arrest and caspase-dependent apoptosis in human lung and gastric cancer, as well as murine melanoma [9,11,12]. However, it remains largely unclear whether inhibition of GSK3β is essential for the anticancer effect of xanthatin. To further reveal potential mechanisms for appropriate coordination of multiple pathways that inactivation of GSK3β by xanthatin dose not readily maintain β-Catenin/Wnt, we address signal transducer and activator of transcription 3 (STAT3), because there is an expansive evidence of literature deciphering that STAT3 regulates a handful of downstream oncogenes shared by β-Catenin. To the best of our knowledge, 1250 overlapping putative target genes have been identified that were co-regulated by β-Catenin/TCF4 and STAT3 [13]. These well-characterized common targets include cell cycle accelerators (c-Myc, CyclinD1, etc.), anti-apoptotic proteins (Bcl-2, XIAP, etc.) and regulators tumor metastasis (COX-2, VEGF, etc.) [14,15]. Actually, STAT3 activation was involved in the nuclear accumulation of β-Catenin, resulting in poor patient survival in colon and breast cancers [16,17]. Thus it is inferred that STAT3 could functionally cooperate with β-Catenin. We therefore hypothesized that disruption of STAT3 might partially attenuate the elevated Wnt/β-Catenin following GSK3β inactivation by xanthatin.

In this study, we examined the effect of xanthatin on STAT3 and GSK3β activities in NSCLC and investigated the underlying crosstalk between STAT3 and Wnt/β-Catenin signalings. The results would sequestrate the doubt of clinical anticancer application of xanthatin in the future.

## Materials and Methods

### Cell culture and cell lines

The human NSCLC lines (A549, H1975, H1650, HCC827) and SV40-immortalized non-tumorigenic human bronchial epithelial cells BEAS-2B used as control were obtained from the Chinese Academy of Sciences Cell Bank of Type Culture Collection (CBTCCCAS, Shanghai, China). The lung cancer cell lines were cultured as monolayers in RPMI 1640 culture media supplemented with 10% fetal bovine serum (Wisent, Quebec, Canada), 100 μg/mL penicillin, and 100 μg/mL streptomycin and BEAS-2B was cultured in serum-free LHC-8 medium (Invitrogen, Carlsbad, CA) maintained in an incubator with a humidified atmosphere of 95% air and 5% CO_2_ at 37°C.

### Chemicals and regents

Xanthatin was isolated and purified by our group as previously described [10] and the purity exceeded 95% as determined by a HPLC method. The 100 mM stock of xanthatin solution was prepared in 100% DMSO and cells treated with equal amount of DMSO served as control. Lithium chloride (LiCl), SB216763 and N-acetyl cysteine (NAC) were purchased from Sigma (St. Louis, MO, USA). Recombinant human IL-6 was obtained from R&D Systems (Minneapolis, MN, USA).

### One solution cell proliferation assay

The cell viability was determined by CellTiter 96^®^ AQueous One Solution cell proliferation assay (Promega, Madison, WI, USA). Briefly, cells were seeded in 96-well cell culture plates and treated with indicated agents. After incubation for indicated time period, 20 μL of One Solution reagent were added to each well and incubation was continued for additional 1-4 h. The absorbance was measured at 490 nm using Synergy™ HT Multi-Mode Microplate Reader (Bio-Tek, Winooski, VT, USA). The effect of indicated agents on cell viability was assessed as the percent of cell viability compared with vehicle-treated control cells, which were arbitrarily assigned 100% viability. The concentration of xanthatin resulting in 50% inhibition of control growth (IC_50_) was calculated by SPSS statistics software. Images of the cell morphology were taken with an inverted microscope (CarlZeiss, Hallbergnoos, Germany) at random fields.

### Flow cytometry assay

Cells were seeded in 6-well cell culture plates and treated with various agents for indicated time period. For cell cycle analysis, cells were detached with trypsin and washed with cold PBS. Precipitated cells were fixed by 500 μL cold 70% ethanol overnight at -20°C. After being washed in PBS, fixed cells were then incubated with RNase at 37°C for 30 min and stained with propidium iodide (PI) for 15 min at room temperature in dark and immediately analyzed by flow cytometry (FACS Calibur, BD Biosciences, San Jose, CA). For cell apoptosis analysis, cells were detached with trypsin and washed with cold PBS. Resuspended cells in 500 μL binding buffer were double stained with FITC-conjugated Annexin V and PI. After 15 min of incubation at room temperature in dark, samples were immediately analyzed by flow cytometry.

### Western blot analysis

Whole-cell lysates were prepared with RIPA buffer containing protease and phosphatase inhibitors. Nuclear and cytoplasmic cell extracts were prepared using the NE-PER Nuclear and Cytoplasmic Extraction kit (Thermo, Rockford, USA). Equal amounts of cell lysates (25 μg) were loaded on 10% SDS-PAGE and transferred onto PVDF membranes. After membranes were blocked, they were incubated with monoclonal antibody against GSK-3 (1∶500, Signalway Antibody) and phosphor-GSK3β Ser-9 (1∶10000, Epitomics), β-Catenin (1∶5000, Epitomics) and phosphor-β-Catenin Ser-33/37/Thr41 (1∶1000, Cell Signaling Technology), STAT3 and phosphor-STAT3 Tyr705 (1∶1000, Cell Signaling Technology), Akt and phosphor-Akt Ser473 (1∶1000, Cell Signaling Technology), GPADH (1∶5000, Bioworld Technology) and Lamin A/C (1∶5000, Epitomics) followed by incubation with horseradish peroxidase-conjugated IgGs (1∶10000, Bioworld Biotechnology). Target proteins were detected by the ECL system (Millipore, Braunschweig, Germany) and visualized with the ChemiDoc XRS system (Bio-Rad, Hercules, CA, USA).

### Plasmids and gene transfection

Cells were transiently transfected with plasmids (0.1 μg/well for 96 well culture plates and 2 μg/well for 6 well culture plates) containing the hemagglutinin (HA)-tagged constitutively active (S9A) GSK3β (Plasmids 14754, Addgene, Cambridge, MA, deposited by Dr. Jim Woodgett) and pcDNA3.0 empty vector (Invitrogene, Carlsbad CA, USA) as control using the opti-MEM medium (Gibco-BRL/Invitrogen, Carlsbad, CA) plus X-tremeGENE HP DNA transfection reagent (Roche, Mannheim, Germany) according to the manufacturer's recommendations. After 48 h post-transfection, the cells were treated with indicated agents for Western blot analyses and proliferation assay.

### RNA interference

Oligonucleotides for human STAT3 siRNA kit were purchased from OriGene (Rockville, MD, USA). The kit contains three predesigned duplexes targeting a specific gene of interest, and we used a pool of three target siRNAs to ensure work efficiency. Cells were transfected with STAT3 siRNA or non-specific siRNA (0.15 μg/well for 96 well culture plates and 2 μg/well for 6 well culture plates) using the opti-MEM plus X-tremeGENE siRNA transfection reagent (Roche, Mannheim, Germany) according to the instruction manual. After 24 h post-transfection, the cells were further treated with xanthatin for Western blot analyses and proliferation assay.

### Quantitative real-time PCR

Total RNA was isolated using TRIzol reagent (Invitrogen, Carlsbad, CA, USA). First-strand cDNA was synthesized with 1 μg total RNA using a PrimeScript RT reagent kit (TakaraBio, Tokyo, Japan). QRT-PCR was performed using IQ^TM^ SYBR Green supermix and the iQ5 real-time detection system (Bio-Rad Laboratories, Hercules, CA). The comparative cycle threshold (Ct) method was applied to quantify the expression levels through calculating the 2^(-∆∆Ct)^ method. The primers used for PCR were as follows (sense and antisense, respectively): GAPDH: cgagatccctccaaaatcaa and ttcacacccatgacgaacat; Cyclin D1: gtgctgcgaagtggaaacc and atccaggtggcgacgatct; c-Myc: taccctctcaacgacagcag and tcttgacattctcctcggtg; Bcl-2: gtggagagcgtcaaccgggaga and gggccgtacagttccacaaaggc; XIAP: cgagcagggtttcttttatactgg and tgtagactgcgcgtggcact. cDNAs amplification and relative expression values were obtained from three independent experiments.

### Immunocytochemistry

After A549 cells on glass coverslips were treated by indicated agents, they were fixed by pre-cold acetone, then rinsed three times with PBS. The cells were permeabilized in 0.1% Triton X-100 and incubated with 1% BSA/PBS to block nonspecific binding. Subsequently, the cells were immunostained by incubating with rabbit monoclonal antibody against β-Catenin (diluted 1∶500, Epitomics) overnight at 4°C. After being washed with PBS, cells were incubated with FITC-conjugated goat anti-rabbit secondary antibody (diluted 1∶60, Boster Biotechnology, Wuhan, China). Nuclei were counterstained with Hoechst 33258 (Biotime Biotech, Haimen, China). Images were taken and analyzed using the ZEN 2011 imaging software on a Zeiss invert microscope (CarlZeiss, Hallbergnoos, Germany) under 400-fold magnification.

### Dual luciferase reporter gene assay

Cells in 96 well culture plates were transiently transfected with 0.1 μg/well p-STAT3-TA-Luc reporter plasmids (Biotime Biotech, Haimen, China) or TCF/LEF-responsive luciferase (Luc2P/TCF-LEF/Hygro, Promega, Madison, WI, USA). Transfection efficiency was normalized with renilla luciferase reporter plasmids. After 18 h post-transfection, cells were treated with indicated agents. Relative promoter activity was measured by dual-luciferase reporter (DLR) assay system using the Glomax 96 Microplate Luminometer (Promega, Madison, WI, USA). For co-transfection experiments, cells in 96 well culture plates were co-transfected with STAT3 siRNA (0.08 μg/well), target and control reporter plasmids (0.15 μg/well in total) for 24 h and then treated with indicated agents, then underwent DLR assay.

### Statistical analysis

The data were presented as mean ± SD. Differences in the results of two groups were evaluated using either two-tailed Student's t test or one-way ANOVA followed by *post hoc* Dunnett's test. The differences with *P*<0.05 were considered statistically significant.

## Results

### Xanthatin inhibits the proliferation of NSCLC

**Figure 1:**
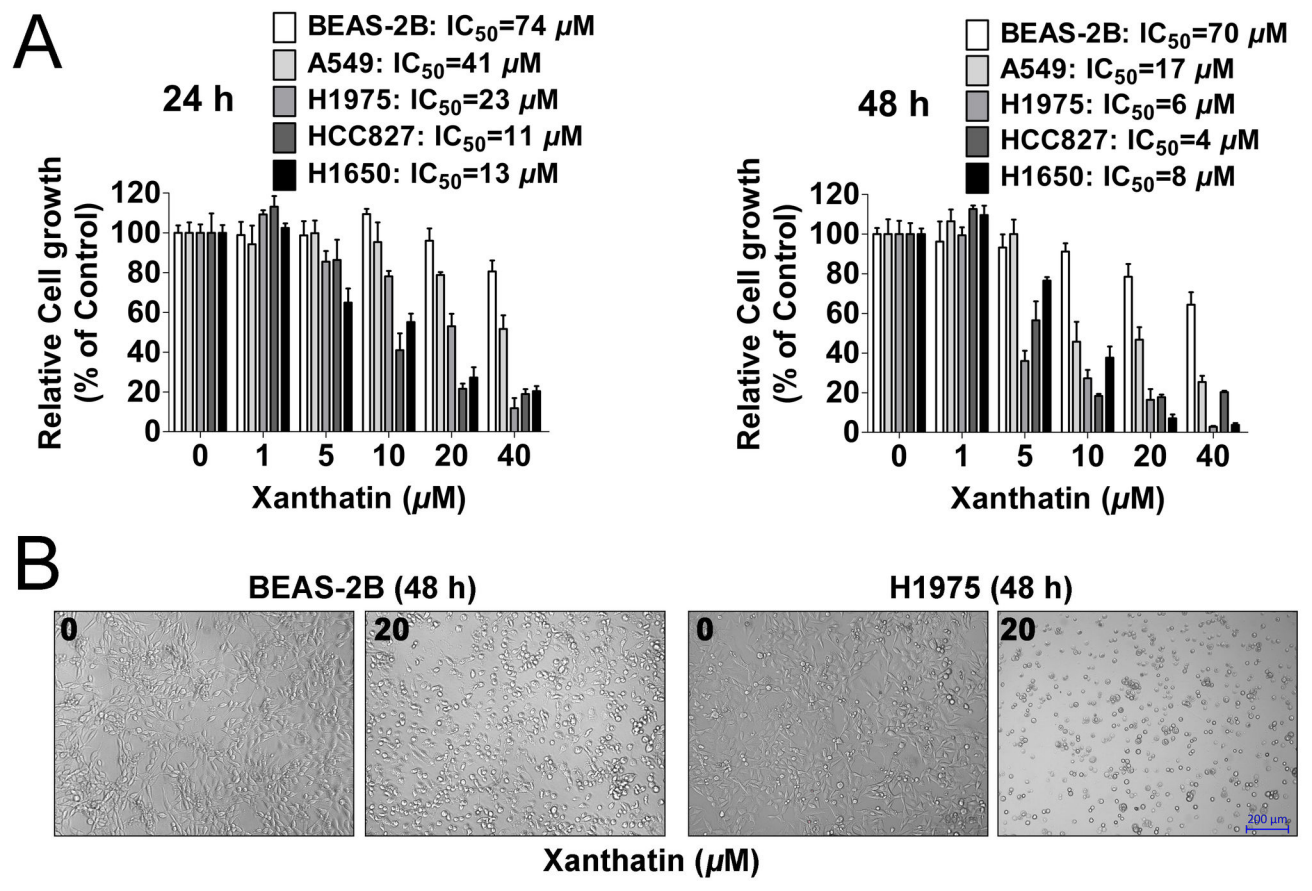
*In* *vitro* treatment of human NSCLCs with xanthatin inhibits the proliferation potential in a dose- and time-dependent manner. (A) NSCLCs (A549, H1975, HCC827, H1650 cells) and human bronchial epithelial cells (BEAS-2B) were exposed to indicated concentrations of xanthatin (1, 5, 10, 20, 40 μM) for 24 h and 48 h, respectively. Cell viability was determined by One solution cell proliferation assay. The data are presented as mean ± SD. The values are expressed as percentage of viable cells normalized to percentage of viable cells in 0.5% DMSO-treated cells. The concentration of xanthatin resulting in 50% inhibition of control growth (IC_50_) was calculated by SPSS statistics software using Probit model. (B) Representative images of cell morphology in BEAS-2B and H1975 cells were taken after 48 h treatment with or without 20 μM xanthatin (100×, scale bar represents 200 μm).

To access the anticancer activities of xanthatin, four human NSCLC cell lines A549, H1975, H1650 and HCC827, as well as normal (non-neoplastic) human bronchial epithelial cells BEAS-2B were used. As shown in Figure 1A, we found xanthatin inhibited NSCLC proliferation in a dose and time responsive manner. IC_50_ values from each cancer cell line and incubation time were calculated, showing that xanthatin exerted 50% inhibition under 20 μM after 48 h treatment. We also noted the inhibitory effect on normal bronchial epithelial cells kept at high micromolar concentrations than the effect of equivalent doses and incubation time of xanthatin in NSCLC. Representative images of cell morphology showed that xanthatin selectively killed tumor cells rather than normal cells (Figure 1B). Collectively, these data demonstrate that xanthatin has universal anticancer activity in NSCLC without apparent physical toxicity.

### Xanthatin preferentially inhibits the activation of STAT3 rather than GSK3β in NSCLC

**Figure 2:**
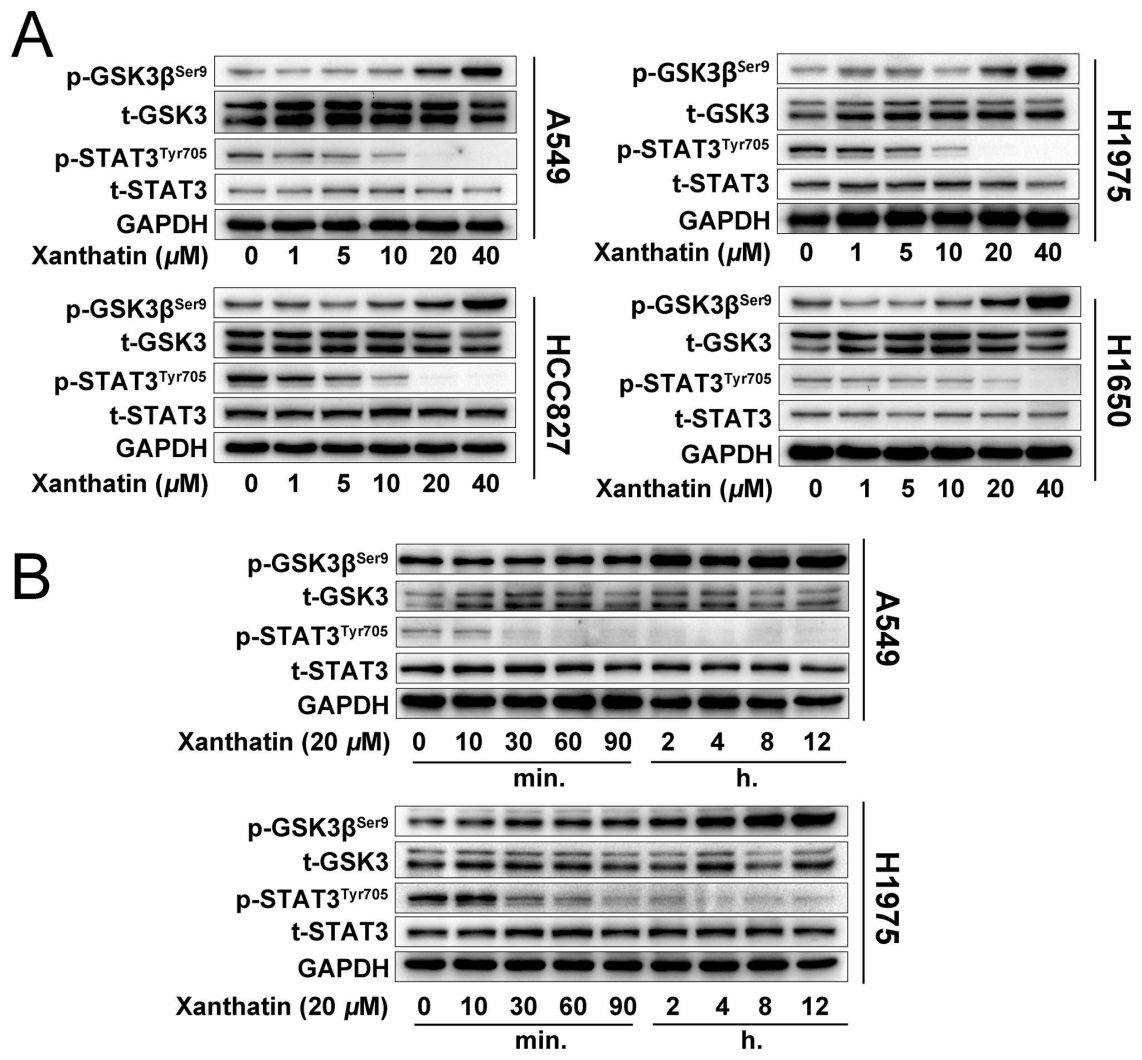
Xanthatin suppresses STAT3 and GSK3β signaling of human NSCLCs in a dose- and time-dependent manner. (A) NSCLCs (A549, H1975, HCC827, H1650 cells) were treated with indicated concentrations of xanthatin (1, 5, 10, 20, 40 μM) for 6 h and then were subjected to Western blot for measuring protein levels of phosphor-GSK3β (Ser9), t-GSK3, phosphor-STAT3 (Tyr705) and STAT3 respectively. (B) A549 and H1975 cells were treated with 20 μM xanthatin for various time (0-12 h) and then were subjected to Western blot for measuring protein levels of phosphor-GSK3β (Ser9), GSK-3, phosphor-STAT3 (Tyr705) and STAT3 respectively.

We next evaluated whether downregulation of GSK3β and STAT3 activity was concurrent with the anticancer effects of xanthatin. We treated NSCLC with various concentrations of xanthatin (1, 5, 10, 20 and 40 μM) for 6 h, or 20 μM xanthatin within 12 h. Western blot analysis showed that xanthatin dose-dependently increase the phosphor-GSK3β (Ser9) and decrease the phosphor-STAT3 (Tyr705) without total protein level changed in A549, H1975, H1650 and HCC827 cells (Figure 2A). Additionally, we observed that xanthatin potently abrogated STAT3 activity in 30 min, but phosphorylated GSK3β began to increase after 2 h treatment in A549 and H1975 cells (Figure 2B), suggesting that xanthatin primarily suppressed STAT3 instead of GSK3β. Collectively, these results suggest that inactivation of both GSK3β and STAT3 is involved in the cellular events triggered by xanthatin.

### Inactivation of GSK3β and STAT3 are two mutually exclusive events in xanthatin-induced cell death

**Figure 3:**
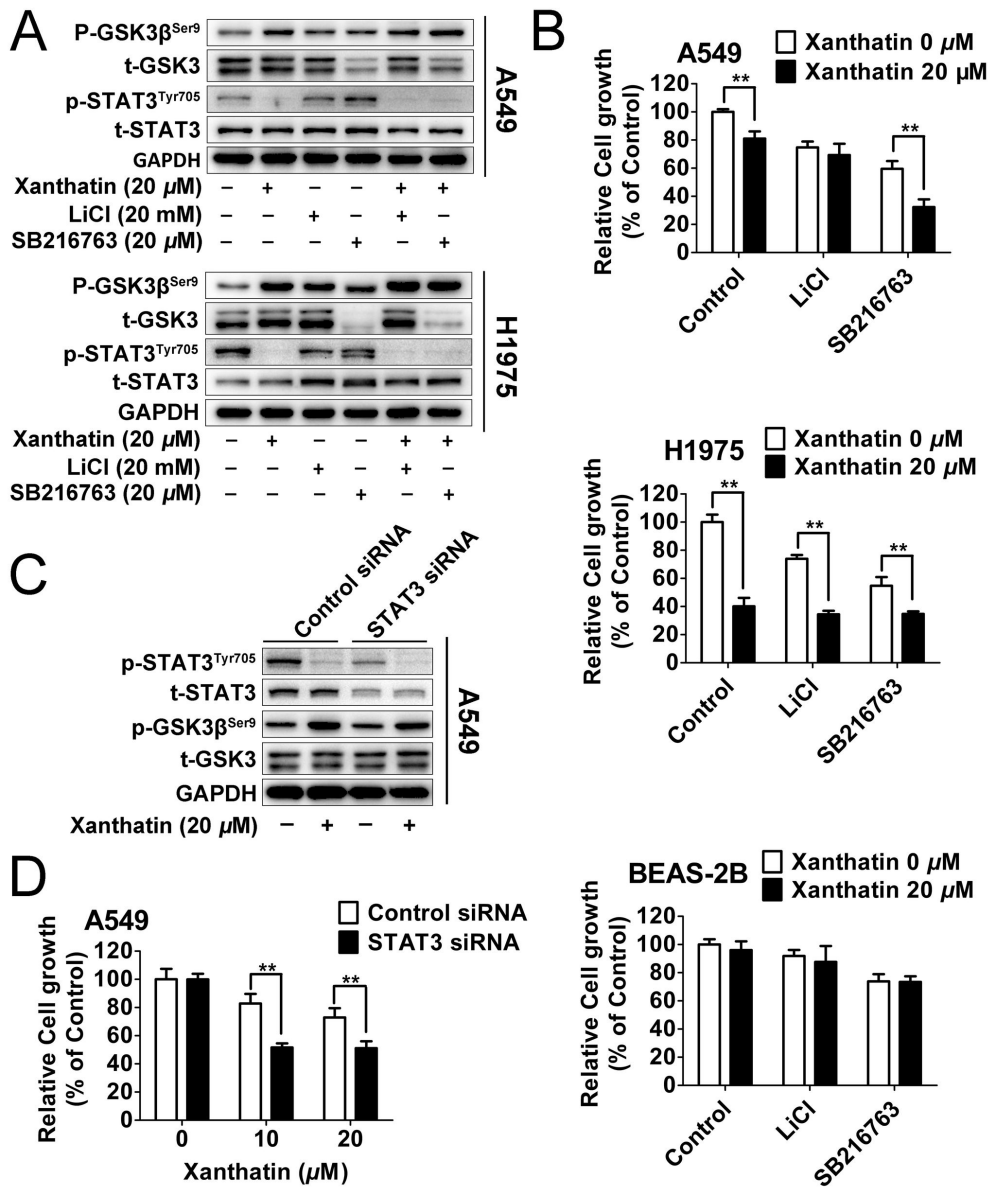
Inactivation of GSK3β and STAT3 are two mutually exclusive events in xanthatin-induced cell death. (A) A549 and H1975 cells were treated with 0.5% DMSO or 20 μM xanthatin co-incubated with or without 20 mM LiCl or 20 μM SB216763 for 6 h. Protein samples were subjected to Western blot for measuring phosphor-GSK3β (Ser9), GSK-3, phosphor-STAT3 (Tyr705) and STAT3. (B) NSCLC (A549, H1975 cells) and BEAS-2B were exposed to 0.5% DMSO or 20 μM xanthatin co-incubated with or without 20 mM LiCl or 20 μM SB216763 for 24 h. Cell viability was determined. The data are presented as mean ± SD (For indicated comparisons, *P<0.05, **P<0.01). (C) Control siRNA or siRNA against STAT3 were transfected into A549 cells (2 μg siRNA per well). After 24 h post transfections, cells were treated with with or without 20 μM xanthatin for 6 h following with Western blot for measuring phosphor-GSK3β (Ser9), GSK-3, phosphor-STAT3 (Tyr705) and STAT3. (D) Control siRNA or siRNA against STAT3 were transfected into A549 cells (0.15 μg siRNA per well). After 24 h post transfections, cells were treated with with 10 or 20 μM xanthatin for further 24 h and subjected to cell proliferation assay. The data are presented as mean ± SD. For indicated comparisons, *P<0.05, **P<0.01.

To confirm the relationship between the two molecular events caused by xanthatin and potential mechanisms underlying the susceptibility of xanthatin-induced cell death, we examined whether the well-known GSK3β-specific kinase inhibitors, LiCl (non-ATP-competitive inhibitor) and SB216763 (ATP-competitive inhibitor) [18] could generate similar anticancer activity and enhance the anticancer response of xanthatin. As shown in Figure 3A, we found both of 20 mM LiCl and 20 μM SB 216763 increased GSK3β inactivation and the effect significantly strengthened by xanthatin. Interestingly, unlike LiCl, SB216763 could not only increase the phosphorylation of basal GSK3β but also the degradation of total GSK-3 in A549 and H1975 NSCLC, which was out of our expectation. However, pharmacological GSK3β inhibitors failed to influence the level of phosphor-STAT3. To further investigate whether GSK3β inactivation might have impact on cell survival, we found that the proliferation of A549 and H1975 cells was markedly inhibited by GSK3β inhibitors alone or in combination with xanthatin for 24 h treatment, and normal human bronchial epithelial cells were weakly affected by GSK3β inhibitors with or without xanthatin (Figure 3B).

We also used siRNA to curb STAT3 function in A549 cells as an extensive validation. Western blot analysis confirmed significant suppression of phosphor-STAT3 and total STAT3 expression by target siRNA, which generated additive inhibition of STAT3 activity in the presence of xanthatin. However, STAT3 siRNA also failed to alter GSK3β activity compared to cells transfected with control siRNA (Figure 3C). Additionally, silencing of STAT3 enhanced the ability of xanthatin to reduce the growth of A549 cells after 24 h treatment (Figure 3D). Altogether, it is plausible to conclude that blockage of GSK3β and STAT3 activities is implicated in the antiproliferative effects of xanthatin, but the two events are not causally connected.

### Pharmacological inhibition of GSK3β partially potentiates xanthatin-induced cell arrest and apoptosis

**Figure 4:**
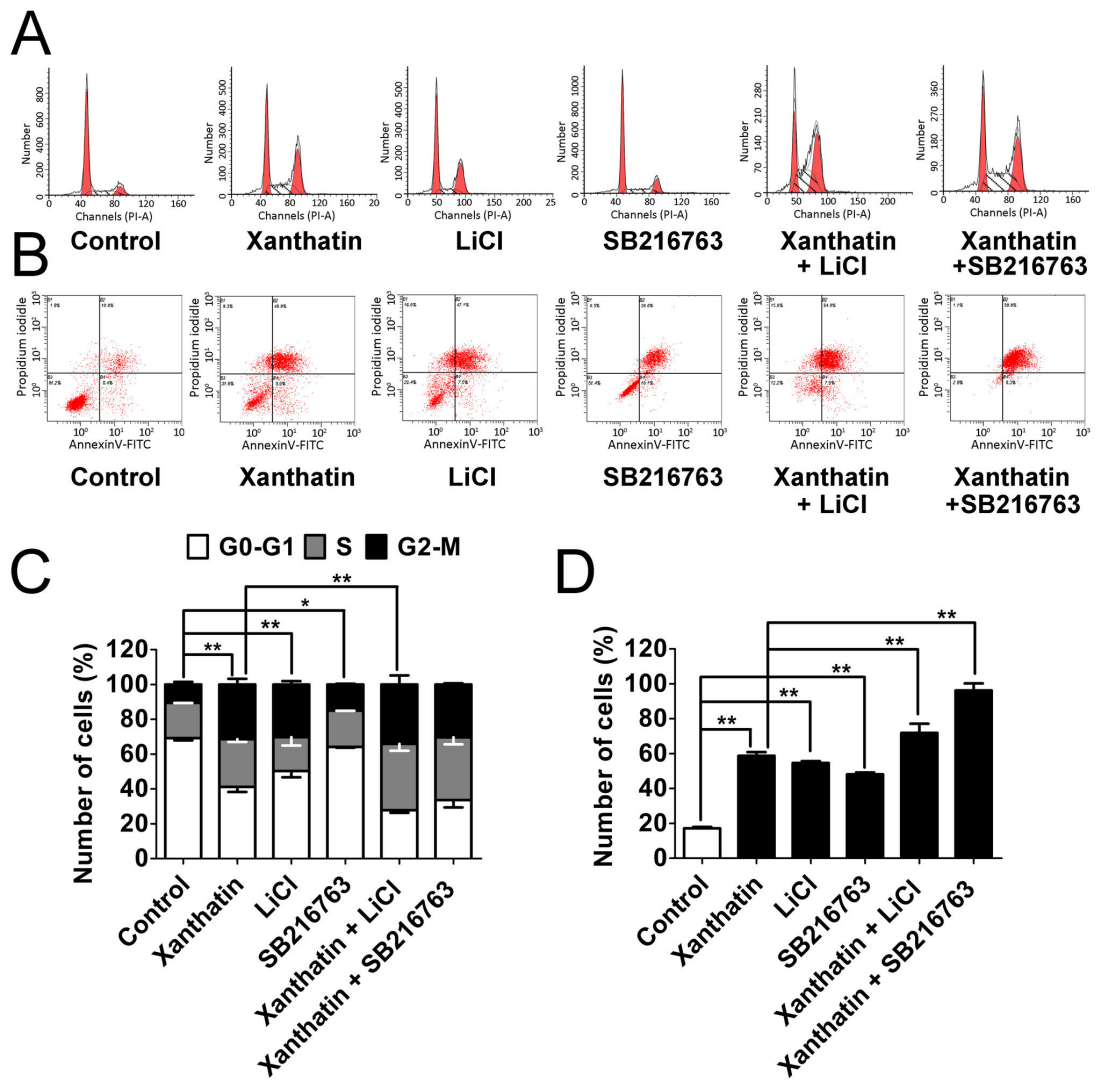
Pharmacological inhibition of GSK3β partially potentiates xanthatin-induced cell cycle arrest and apoptosis in A549 cells. (A) A549 cells were exposed to 0.1% DMSO or 20 μM xanthatin co-incubated with or without 20 mM LiCl or 20 μM SB216763 for 24 h and then stained with propidium iodidle for detecting the cell cycle by flow cytometry. (B) A549 cells were exposed to 0.1% DMSO or 20 μM xanthatin co-incubated with or without 20 mM LiCl or 20 μM SB216763 for 48 h and then stained with propidium iodidle and AnnexinV-FITC for detecting the apoptosis by flow cytometry. (C) Quantitative data of cell cycle (G0-G1, S, and G2/M phases) are drawn from average of triplicate determinations and shown as mean ± SD. For indicated comparisons, *P<0.05, **P<0.01. (D) Total percentages of apoptotic cells were summarized by the lower-right quadrant of the fluorescence-activated cell sorting histograms (percentage of early apoptotic cells) and the upper-right quadrant (percentage of late apoptotic cells). The data are shown as mean ± SD. For indicated comparisons, *P<0.05, **P<0.01.

To confirm the enhanced anticancer effects by anti-GSK-3 in xanthatin-exposed cells was due to unbalanced cell proliferation or apoptosis, we then monitored cell cycle arrest and apoptosis responses by flow cytometric analysis in A549 cells. As shown in Figure 4A and 4C, 20 μM xanthatin and 20 mM LiCl could strongly induce cells to undergo G2/M arrest after 24 h treatment (from 10.56-31.42 % and 10.56-30.27 %, respectively), which was accompanied by a decrease in G1 phase (from 69.26-41.21 % and 69.26-50.33 %, respectively). Unlike LiCl, 20 μM SB216763 less distinctly affected G2/M distribution in A549 cells (from 10.56-15.07 %), which was parallel with the previous report [8]. When we co-incubated xanthatin with the two GSK3β inhibitors, significant increase in cells at G2/M phase appeared by LiCl but not SB216763. Cells were also stained with Annexin V/PI for apoptosis analysis. A549 cells exhibited a clear apoptosis after prolonged exposure (48 h) by xanthatin (58.8%), LiCl (54.6%), and SB216763 (48.1%) respectively. Moreover, xanthatin combined with SB216763 augmented more apoptotic cells than LiCl (96.2% versus 71.9%) (Figure 4B and 4D). Taken together, inhibition of GSK3β is probably responsible for xanthatin-induced cell cycle arrest and apoptosis.

### GSK3β inhibition is essential for anticancer activity of xanthatin

**Figure 5:**
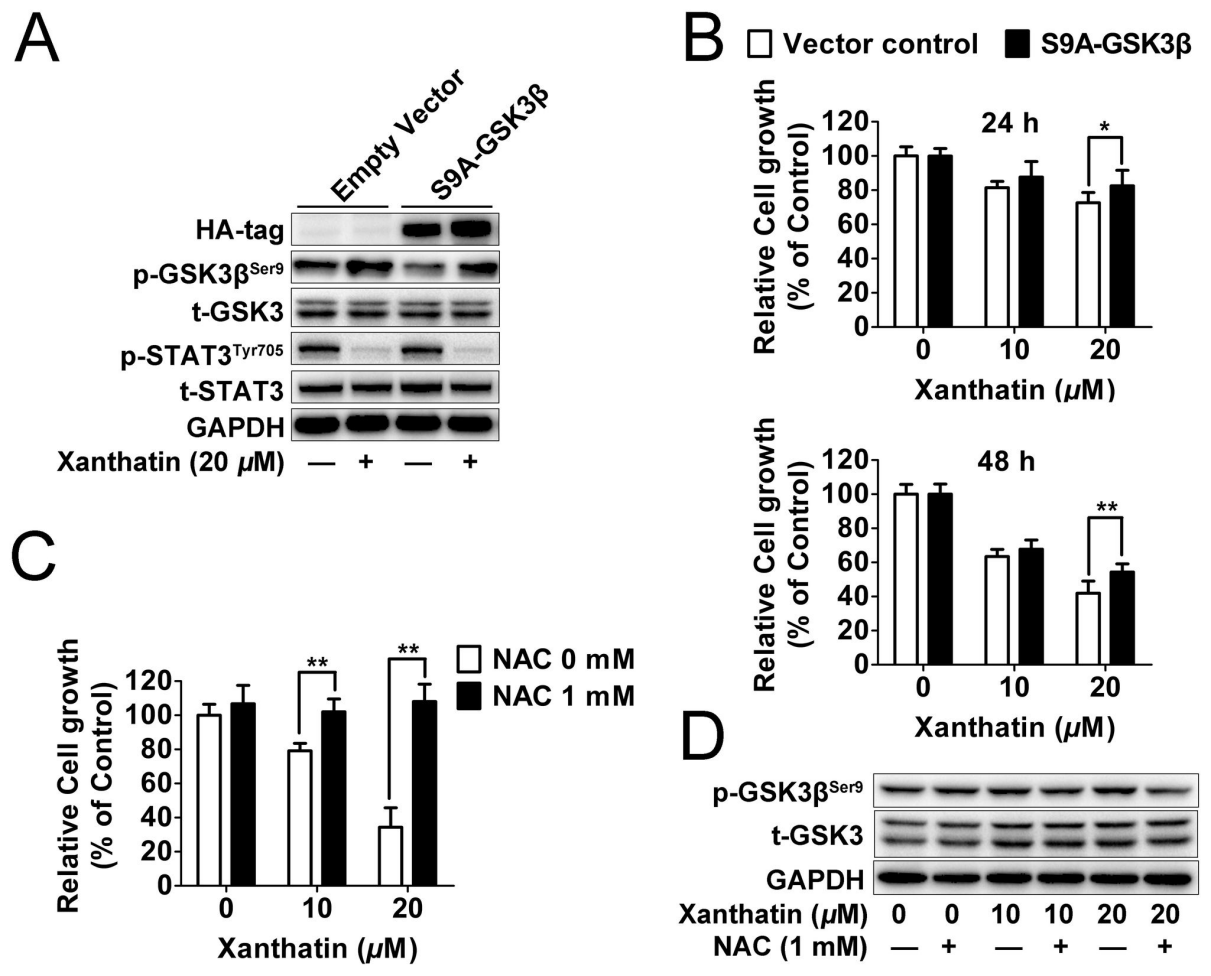
GSK3β is essential for the anticancer effect of xanthatin in A549 cells. (A) A549 cells seeded in 6 well plate were transfected with HA-tagged constitutively active (S9A)-GSK3β or empty vector (2 μg plasmid DNA per well). After for 48 h post transfections, cells were treated with or without 20 μM xanthatin for 6 h, then were subjected to Western blot for measuring protein levels of HA tag, phosphor-STAT3 (Tyr705) and STAT3 respectively. (B) A549 cells seeded in 96 well plate were transfected with HA-tagged constitutively active (S9A)-GSK3β or empty vector (0.1 μg plasmid DNA per well). After for 48 h post transfections, cells were treated with 10 or 20 μM xanthatin for 24 h and 48 h respectively, then were subjected to cell proliferation assay. For indicated comparisons, *P<0.05, **P<0.01. (C) A549 cells were treated with 10 or 20 μM xanthatin in presence or absence of 1 mM NAC for 48 h, then were subjected to cell proliferation assay. (D) A549 cells were treated with 10 or 20 μM xanthatin in presence or absence of 1 mM NAC for 6 h, then were subjected to Western blot for measuring protein levels of phosphor-GSK3β (Ser9) and GSK-3 respectively. The data shown are represented as the mean ± SD. For indicated comparisons, *P<0.05, **P<0.01.

To directly study whether inactivation of GSK3β correlated with xanthatin-induced cell death, we transiently transfected A549 cells with a constitutively active (S9A) construct of GSK3β. As we found, ectopic expression of constitutively active GSK3β attenuated the phosphorylation of GSK3β by xanthatin, but still failed to alter the levels of STAT3 signaling in A549 cells (Figure 5A), which further indicated that inhibition of GSK3β by xanthatin was not relied upon inhibition of STAT3. As expected, overexpression of constitutively active GSK3β conferred a resistance to the anticancer activities of xanthatin in time-dependent manner (Figure 5B). Besides, GSK3β is profoundly involved in the apoptosis escape and its inactivation is an upstream event of the reactive oxygen species (ROS) generation [19,20]. To this end, we observed that xanthatin-induced cell death was completely prevented in the presence of a powerful antioxidant and ROS scavenger N-acetyl cysteine (NAC) (Figure 5C). Moreover, NAC fully attenuated GSK3β phosphorylation by xanthatin (Figure 5D), which demonstrated the role of GSK3β inhibition by xanthatin in ROS-mediated cell death. Altogether, our data suggest that GSK3β inhibition is essential for the anticancer activity of xanthatin via ROS.

### Xanthatin fails to trigger overall post-transcriptional activity of β-Catenin in response to GSK3β inactivation

**Figure 6:**
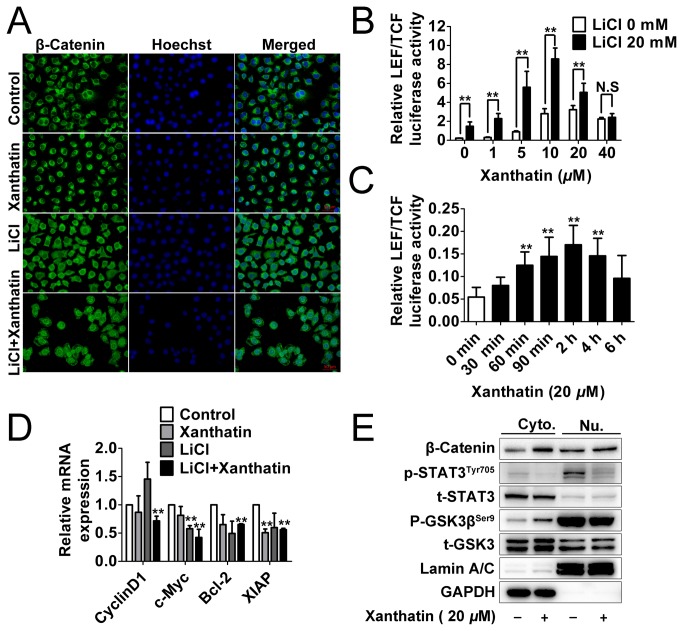
Xanthatin fails to trigger overall post-transcriptional activity of β-Catenin in response to GSK3β inactivation. (A) A549 cells were treated with vehicle, 20 μM xanthatin, 20 mM LiCl or 20 mM LiCl plus 20 μM xanthatin for 12 h, then cells were fixed and incubated with primary antibodies against β-Catenin. A549 cells were immunostained with anti-rabbit FITC-conjugated secondary antibody and then stained with Hoechst 33258. The specimens were visualized and photographed using a fluorescence microscope (400×, scale bar represents 50 μm). Blue depicts the nucleus and green depicts localization of β-Catenin. (B) A549 cells grown to 70-90% confluence were co-transfected with Luc2P/TCF-LEF/Hygro and renilla luciferase (0.1 μg plasmid DNA per well in total) for 18 h, then were sitimulated with vehicle, 20 mM LiCl or 20 mM LiCl plus 1, 5, 10, 20 and 40 μM xanthatin for 6 h. The cell lysates were performed by DLR assay, and the ratio of firefly luciferase to Renilla (relative luciferase) activity was determined. For indicated comparisons, *P<0.05, **P<0.01. N.S, non-significant. (C) A549 cells grown to 70-90% confluence were co-transfected with Luc2P/TCF-LEF/Hygro and renilla luciferase (0.1 μg plasmid DNA per well in total) for 18 h, then were stimulated with 20 μM xanthatin for 0-6 h. The cell lysates were performed by DLR assay, **P<0.01 compared with the control group. (D) A549 cells were treated with or without 20 μM xanthatin for 12 h and then total RNAs were extracted. CyclinD1, c-Myc, Bcl-2, XIAP mRNA levels were determined by means of quantitative real-time PCR and normalized to the level of GAPDH mRNA. The fold changes of mRNA expression of indicated genes were compared as a ratio to the vehicle control. The data are shown as mean ± SD of triplicates experiments. **P<0.01 compared with the control group. (E) A549 cells were treated with or without 20 μM xanthatin for 6 h. Cytoplasmic (Cyto.) and nuclear (Nu.) extracts were prepared and then subjected to Western blot for measuring protein levels of β-Catenin, phosphor-GSK3β (Ser9), GSK-3, phosphor-STAT3 (Tyr705) and STAT3 respectively.

To thoroughly assess the possibility that xanthatin might cause deleterious consequences due to GSK3β inhibition via upregulating β-Catenin mediated transcription activity and oncogenes expression, we set out to examine the intracellular signaling cascade in response to the stimulation of xanthatin and GSK3β inhibitor. As illustrated in Figure 6A, treatment with 20 μM xanthatin for 12 h hardly induced broad nuclear translocation of β-Catenin, while LiCl alone rendered β-Catenin stability and general nuclear distribution. Co-incubation of the two agents caused most serious incident, but the cell number was greatly decreased. Then, we studied the alteration of promoter activity by activated β-Catenin using luciferase construct with LEF/TCF response element, which was implicated in transcription DNA binding of β-Catenin for studying Wnt activation. We found that xanthatin dose-dependently increased (0-10 μM) but decreased (20-40 μM) the pre-transcriptional activity of Wnt/β-Catenin with or without 20 mM LiCl after 6 h incubation (Figure 6B). We further investigated the time course kinetics characterization of 20 μM xanthatin on β-Catenin-LEF/TCF luciferase activity. Xanthatin induced a time dependent activation of the reporter gene within 2 h but after that, the maximum effect began to decrease within 6 h. These data confirm that xanthatin has the possibility to activate β-Catenin/Wnt, but this incident is not sustained.

Next, we determined the mRNA levels of four representative Wnt target genes by quantitative real-time PCR assay. As shown in Figure 6D, Cyclin D1, c-Myc, Bcl-2 and XIAP were unaffected or even repressed by xanthatin after 12 h incubation. Although LiCl led to subtle fold increase of CylinD1, but did not show statistical significance. Additionally, the activity of GSK3β was also regulated by subcellular localization [21], we therefore attempted to explore the effect of xanthatin in different cell fractions. Quite interestingly, xanthatin resulted in increased GSK3β inactivation in cytoplasm but failed or even slightly decreased it in nucleus. The nuclear GSK3β seemed to appear highly inactivated compared to cytoplasmic counterpart (the ratio of phosphorus to total protein from equal mass of loading samples). The fold of nuclear accumulation of β-Catenin also exhibited less in cytoplasm. Nevertheless, the cytoplasmic and nuclear STAT3 phosphorylation in A549 cells was both evidently abrogated by xanthatin (Figure 6E). Altogether, our data suggest that xanthatin can not produce noxious risk of β-Catenin/Wnt at post-transcriptional level.

### Suppression of STAT3 attenuates the risk of GSK3β inhibition-induced β-Catenin/Wnt activation by xanthatin

**Figure 7:**
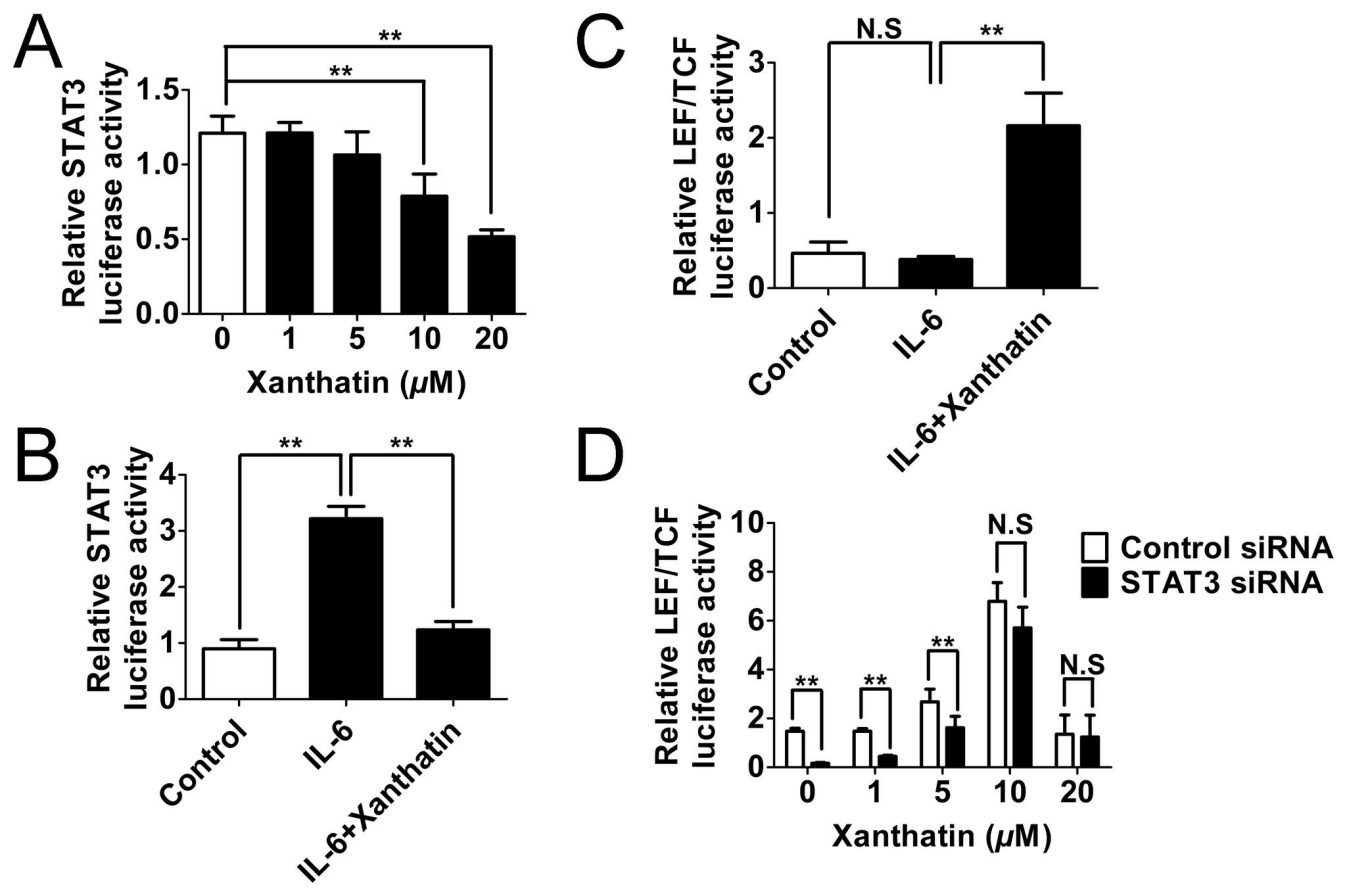
STAT3 inactivation contributes to a reduced risk of the canonical Wnt pathway in xanthatin treated A549 cells. (A) A549 cells grown to 70-90% confluence were co-transfected with p-STAT3-TA-luc and renilla luciferase (0.1 μg plasmid DNA per well in total) for 18 h, then were sitimulated with 1, 5, 10, 20 μM xanthatin for 3 h. (B) A549 cells were co-transfected with p-STAT3-TA-luc and renilla luciferase for 18 h and then sitimulated with 20 ng/mL of IL-6 in absence or presence of 20 μM xanthatin for 3 h. (C) A549 cells were co-transfected with Luc2P/TCF-LEF/Hygro and renilla luciferase for 18 h and then sitimulated with 20 ng/mL of IL-6 in absence or presence of 20 μM xanthatin for 3 h. (D) A549 cells grown to 70-90% confluence were co-transfected with control siRNA or STAT3 siRNA and Luc2P/TCF-LEF/Hygro plus renilla luciferase (0.08 μg siRNA and 0.15 μg plasmid DNA per well in total) for 24 h, then were treated with 1, 5, 10, 20 μM xanthatin for 6 h. All the cell lysates were performed by DLR assay, and the ratio of firefly luciferase to Renilla (relative luciferase) activity was determined. The data are presented as mean ± SD. For indicated comparisons, *P<0.05, **P<0.01. N.S, non-significant.

In view of the potential crosstalk between STAT3 and β-Catenin/Wnt signaling pathways, and together with the above observations, it could be postulated that xanthatin-induced STAT3 inhibition could be prepared to combat potential risk of β-Catenin/Wnt activation. As both activated STAT3 and β-Catenin served as transcription factors, we questioned that whether inhibition of STAT3 activation might deter the functional β-Catenin at the pre-transcriptional level. To this end, we first observed that xanthatin could dose-dependently inhibit basal STAT3 transcriptional activity (Figure 7A). Moreover, we used 20 ng/mL of IL-6 to augment STAT3 signaling, and found that 20 μM of xanthatin strikingly reversed IL-6-induced luciferase activity after 3 h co-incubation (Figure 7B). Unexpected, When we examined whether up-regulation of STAT3 by IL-6 could contribute to an increase in β-Catenin-mediated transcriptional activity, we got negative results (Figure 7C). However, STAT3 silencing was still able to partially attenuate xanthatin-induced LEF/TCF luciferase activity (Figure 7D). Thus we proposed that the risk of Wnt activation mediated by xanthatin might be partially counteracted by STAT3 inhibition.

## Discussion

As early as the 1990s, the cytotoxic activity of xanthatin has been recognized [22] and undergone exploratory study of potential role as a promising natural agent in cancer treatment, yet little is known about its underlying mechanisms and targets. GSK3β has been emerged as a molecular target for cancer therapy [23-25]. In this respect, evidence shows that both chemical inhibitors-caused GSK3β inactivation and small interference RNA-mediated gene silencing are responsible for tumor inhibition [26-29]. Consistent with this point, reports have confirmed that inhibition of cytoplasmic GSK3β can trigger serious cell apoptosis in NSCLC [7,8]. However, more recently, an opposite view proposed that inhibition of GSK3β would increase cisplatin resistance through activation of Wnt/β-Catenin signaling in the same A549 cell line [30]. Whatever, contradiction in this term throws out more light into the double-edged sword in cancer therapy.

In this study, our results could support the notion that inhibition of GSK3β may lead to increased susceptibility to xanthatin-induced apoptotic stimuli in NSCLC. We displayed the anticancer effect of xanthatin in a GSK3β-dependent manner by pharmacological (GSK3β inhibitors) and genetic (constitutively active S9A-GSK3β) validation, suggesting that xanthatin may be a new GSK3β inhibitor. Importantly, both xanthatin and GSK3β inhibitors showed subtle cytotoxicity in normal cells, which further confirmed the high values of the therapeutic target in cancer therapy. Phosphorylation of Ser9-mediated GSK3β inhibition can be carried out by various upstream kinases (such as p70^S6K^, p90^rsk^, protein kinase A (PKA), PKB (Akt), PKC and integrin-linked kinase (ILK) [31-36] and hundreds of proteins are proposed to be substrates for the kinase [37]. How GSK3β inactivation clues by xanthatin from differential upstream regulation need for detailed analysis, and we have excluded that xanthatin inactivated GSK3β via Akt in A549 cells (Figure S1). Therefore, we would also need to gain further insight into the molecular signaling cascades upstream and downstream of GSK3β that are known to be involved in tumorigenesis and xanthatin might interfere with.

The results of our studies prompted us to further elucidate the functional relationship between STAT3 and β-Catenin reflected by xanthatin. Interestingly, the inhibitory effect of xanthatin on STAT3 signaling seemed to be more potent than GSK3β at lower dose, in shorter time and throughout the subcellular fractions. Then we postulated that STAT3 might be an upstream regulator of GSK3β, but STAT3 siRNA did not alter the GSK3β activity regardless of great augmentation of xanthatin-induced cell death. In addition, both of GSK3β inhibitors and expression of constitutively active GSK3β failed to alter the phosphorylation level of STAT3. Thus we speculated that STAT3 might not directly regulate GSK3β, but we still could not rule out a functional crosstalk with GSK3β downstream substrate β-Catenin.

β-Catenin mainly serves as a cytoskeletal structure-associated protein [38] and free ones can be captured by adenomatous polyposis coli (APC) and undergo conventional GSK3β-dependent proteasomal degradation (GSK3β destabilizes β-Catenin by phosphorylating it at Ser33/37/Thr41 [39]. APC mutations are present but infrequent in human lung cancer [40,41], and we detected the intracellular localization of β-Catenin mainly on cytoplasmic membrane and cytoplasm in rest A549 cells. Importantly, xanthatin did not lead to theoretical transcriptional switch of Wnt oncogenes in response to nuclear accumulation of β-Catenin followed by GSK3β inhibition in our study. Previous evidence implied that pro-apoptotic stimuli could induce nuclear accumulation of GSK3β [42] and nuclear GSK3β also inhibited the canonical Wnt signaling pathway in a β-Catenin phosphorylation-independent manner [43]. Thus, our observations indicated that xanthatin inhibited cytoplasmic GSK3β but not the nuclear compartment, which could partially account for the finding that xanthatin eventually failed to stimulate endogenous transcription of Wnt target genes due to a yet unknown nuclear GSK3β substrate that in turn inhibited the Wnt pathway. Assays for transcriptional activity based on LEF/TCF responsive reporter gene showed that upregulation of Wnt/β-Catenin by xanthatin was transient. Therefore, we speculated that there could be other specific modulators located at the the neighbor of LEF/TCF in the promoter region and co-regulating Wnt target genes.

As a candidate, STAT3 has been proposed by accumulated` evidence as mentioned in the introduction. Previous reports showed that p-STAT3 and β-Catenin could not directly interact, while inhibition of STAT3 resulted in the loss of β-Catenin in the nucleus and reduction of β-Catenin/TCF transcription in colorectal cancer [17]. We also found that STAT3 silencing rescued xanthatin-induced β-Catenin/TCF transcription. However, we noted that the upstream specific cytokine IL-6 successfully activated STAT3 signaling but failed to correspondingly increase β-Catenin/TCF transcription with or without xanthatin in A549 cells. To deal with the result, we got some clues from another study: the endogenous protein inhibitor of activated STAT3 (PIASy) could repress LEF activity-mediated Wnt-responsive transcription [44]. This implied STAT3 might cooperate with transcriptional activity of β-Catenin independent of the ligand-mediated activation of STAT3 but interruption of negative feed-back loop of STAT3. We also found STAT3 siRNA didn’t influence the total and the phosphorylated (Ser33/37/Thr41) protein levels of β-Catenin (Figure S2), which raises another question that whether STAT3 inactivation by xanthatin might reduce the retention of β-Catenin in nuclei and promote it shuttling from the nucleus to the cytoplasm. The detailed mechanisms involved the interplay between STAT3 and β-Catenin in NSCLC cells induced by xanthatin warrant further investigation.

**Figure 8:**
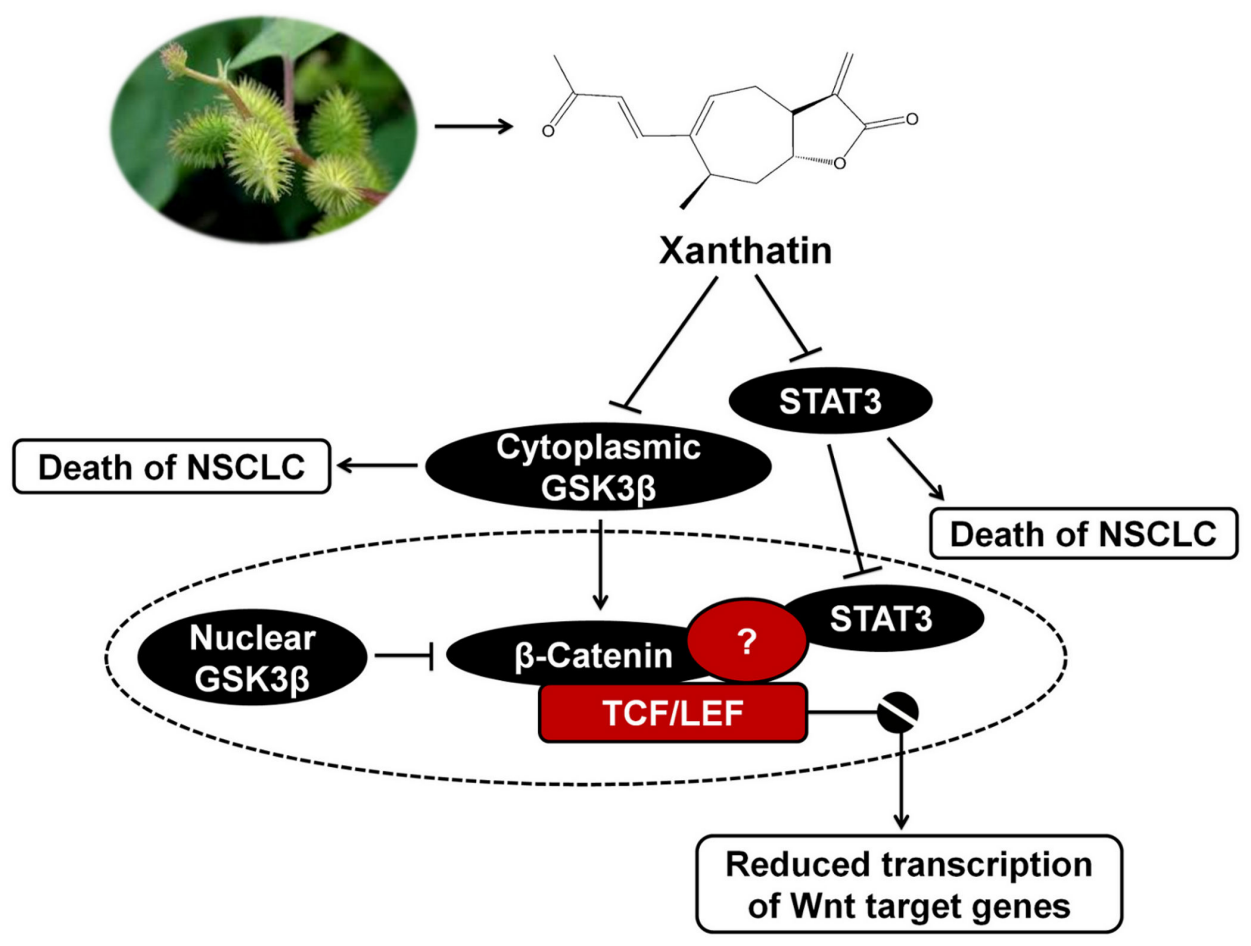
Proposed model by which xanthatin treatment induces cell death in NSCLC.

In summary, the major findings in this study can be illustrated in Figure 8: (1) For the first time, we have confirmed that xanthatin independently downregulate GSK3β and STAT3 activities, which were both essential for the anticancer effect on NSCLC; (2) Dominant STAT3 inhibition contributes to minimize the risk of the canonical Wnt pathway in response to inactive GSK3β, which offers a new perspective on the ability to inhibit more than one pathway interpreted by xanthatin in cancer treatment.

## Supporting Information

Figure S1 **Xanthatin has no effect on Akt signaling in A549 cells.** A549 cells were treated with indicated concentrations of xanthatin (1, 5, 10, 20, 40 μM) for 6 h and then were subjected to Western blot for measuring protein levels of phosphor-Akt (Ser473) and t-Akt respectively.
(TIF)

Figure S2 **Knockdown of STAT3 has no effect on GSK3β-mediated stability of β-Catenin.** Control siRNA or siRNA against STAT3 were transfected into A549 cells (2 μg siRNA per well). After 24 h post transfections, cells were treated with or without 20 μM xanthatin for 6 h following with Western blot for measuring phosphor-β-Catenin (Ser33/37/Thr41) and β-Catenin.
(TIF)
