# Supplementary figures and images for: Concerted Suppression of STAT3 and GSK3β Is Involved in Growth Inhibition of Non-Small Cell Lung Cancer by Xanthatin
Source: PLoS One. 2013 Nov 28;8(11):e81945. doi: 10.1371/journal.pone.0081945 (PMC3842975; doi:10.1371/journal.pone.0081945)

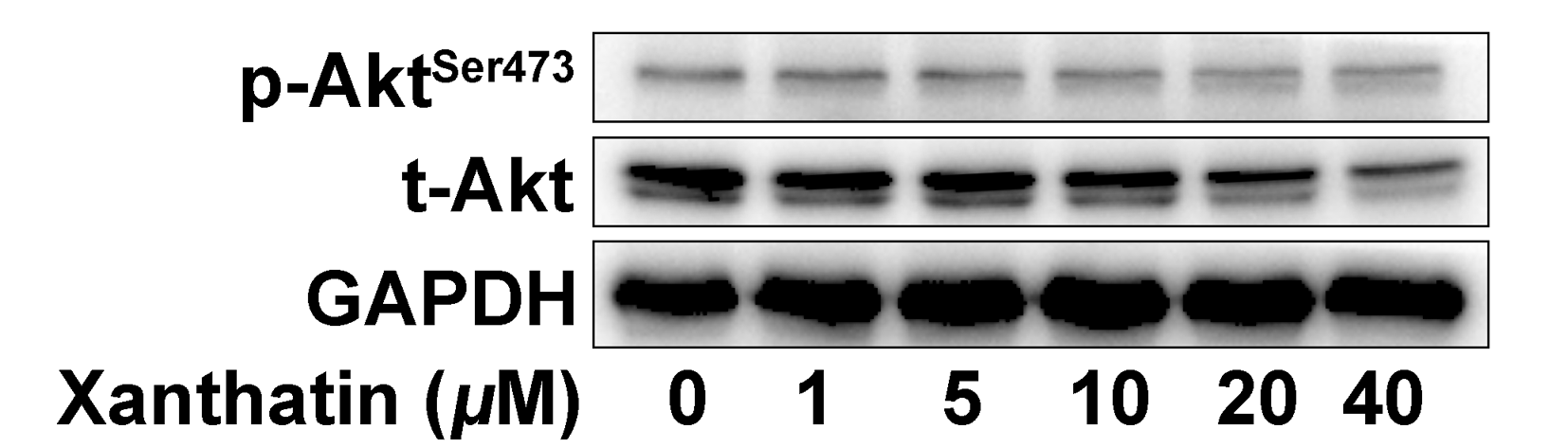

Supplement: Figure S1 — Xanthatin has no effect on Akt signaling in A549 cells. A549 cells were treated with indicated concentrations of xanthatin (1, 5, 10, 20, 40 μM) for 6 h and then were subjected to Western blot for measuring protein levels of phosphor-Akt (Ser473) and t-Akt respectively. (TIF) [file pone.0081945.s001.tif]

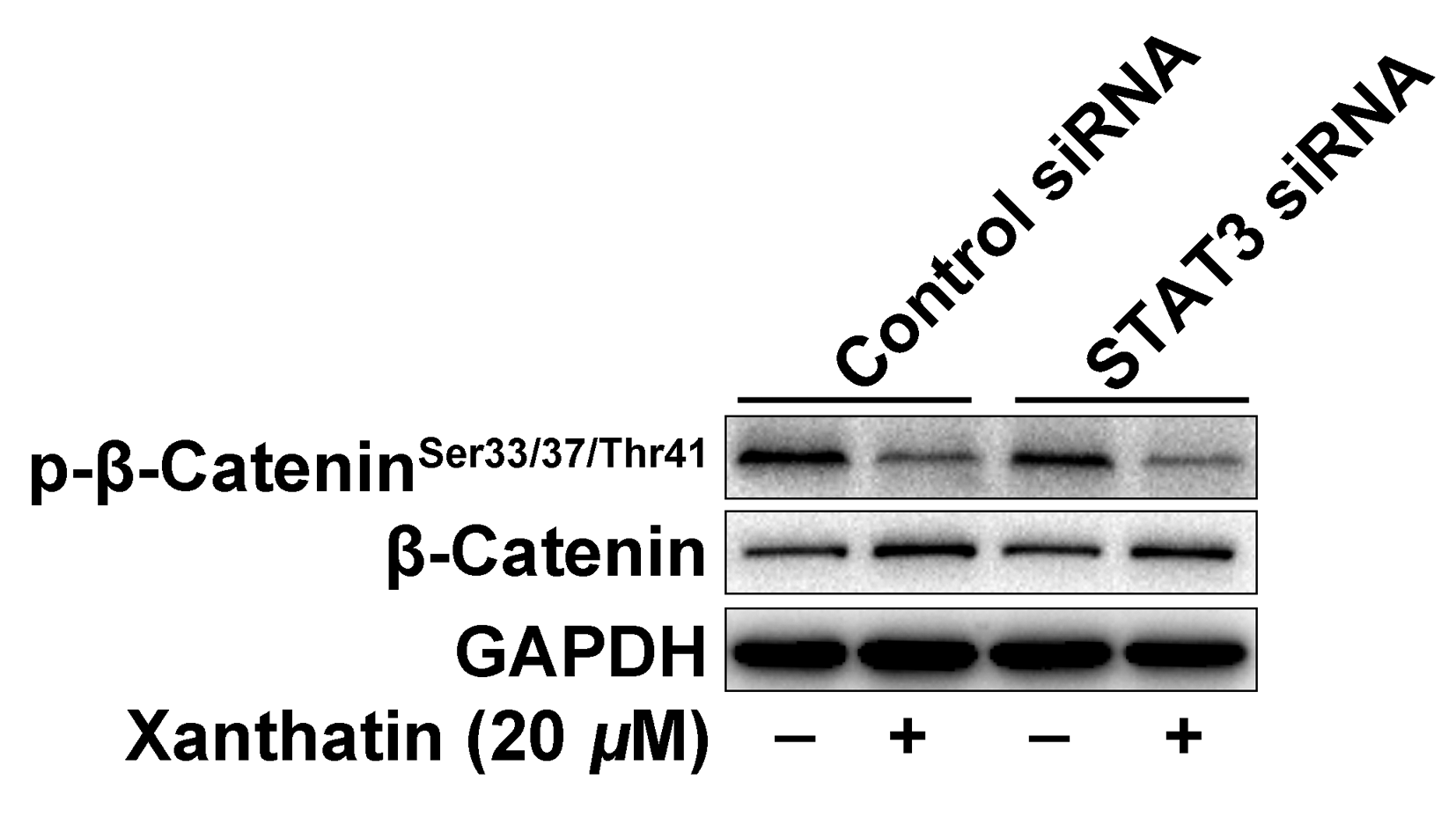

Supplement: Figure S2 — Knockdown of STAT3 has no effect on GSK3β-mediated stability of β-Catenin. Control siRNA or siRNA against STAT3 were transfected into A549 cells (2 μg siRNA per well). After 24 h post transfections, cells were treated with or without 20 μM xanthatin for 6 h following with Western blot for measuring phosphor-β-Catenin (Ser33/37/Thr41) and β-Catenin. (TIF) [file pone.0081945.s002.tif]
